# Supplementary material for: Polymorphisms in Pfkelch13 domains before and after the introduction of artemisinin-based combination therapy in Southwest Nigeria
Source: PLoS One. 2025 Mar 31;20(3):e0316479. doi: 10.1371/journal.pone.0316479 (PMC11957316; doi:10.1371/journal.pone.0316479)
Supplement: Supporting information 2 — (ZIP) [file pone.0316479.s002.zip › 069KN2F_PREMIX_Plate_KELCH2_B06.pdf]

Samples: 11991
Bases: 1057
Average spacing: 12

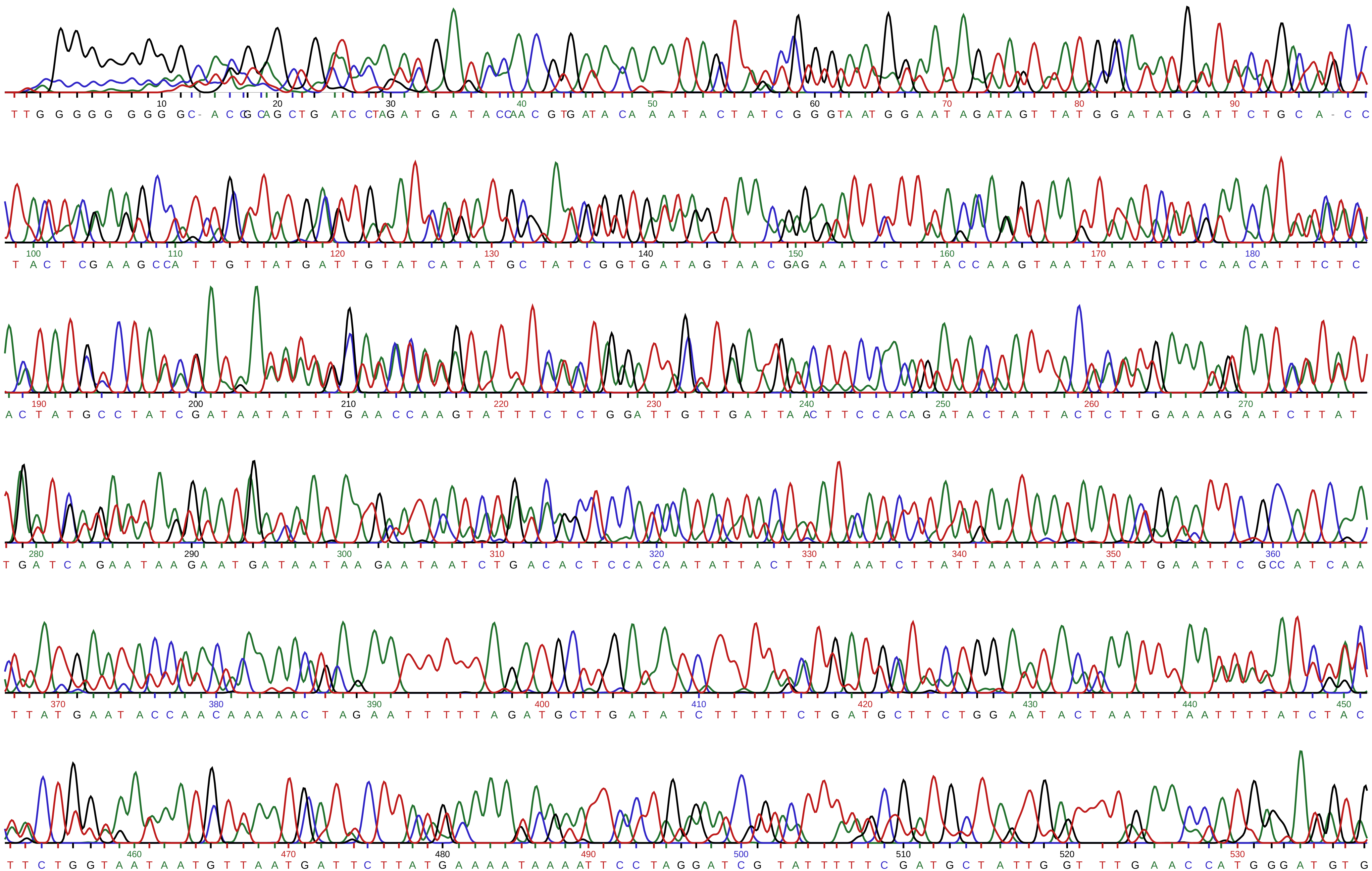

Samples: 11991
Bases: 1057
Average spacing: 12

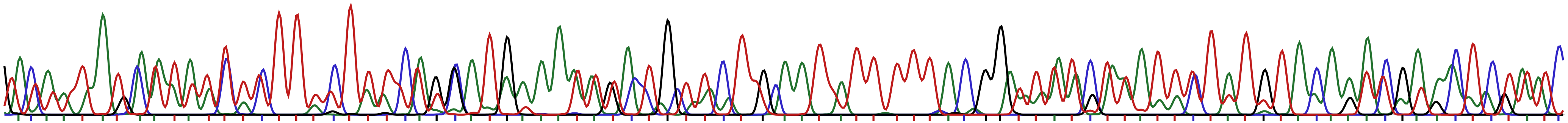

540 550 560 570 580 590 600 610 620

A C A A T A T A A T A T T T C T T T C T T T C A G C A T G A A A T A T C T G T T C T G A A T A T T T T T A C G G T T A T C T T A T T C T A T G T A C A A A C G A A C T C T A T C

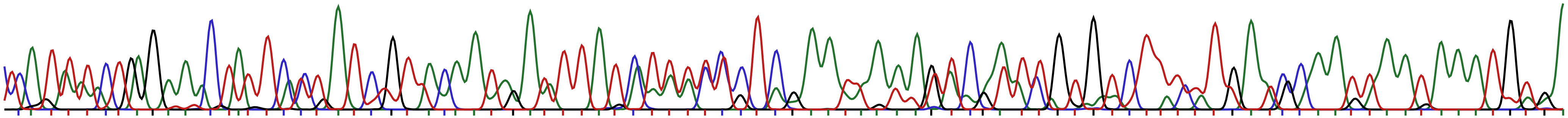

630 640 650 660 670 680 690 700 710

C A T T T C T A G A A C T A T T C C T A T C G T A C A A T G A T T T A T C T T T T C C T C G A A T A A A A G T C G A T T G T G T C T T T T T G A T C C A A T T A A T A A A T G T G A

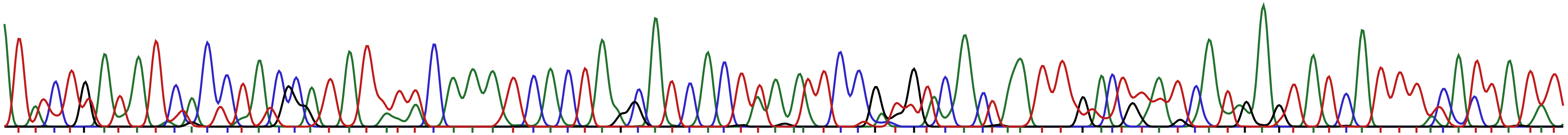

720 730 740 750 760 770 780 790 800

T T C T G A T A T C A C C T A C C A T A T A T T C A A A T C A C T A G C A T C A C T T A A A T C C G T G T C A C T A A T T G A C T T A T C A T G A G T A T C A T T T A C A T T A T A T

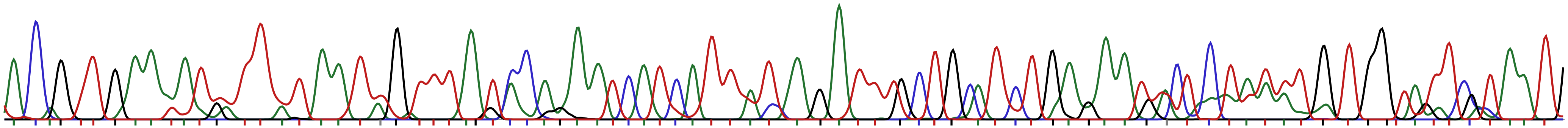

810 820 830 840 850 860 870 880 890

A C A G T T G A A T A T G T T A T A A T - G T T T A G T C C A G A A T C A T C A T T A T A G A T T G C T G A T C T G A G A A G - T C T A T A T G T G G T A T T C T A A T

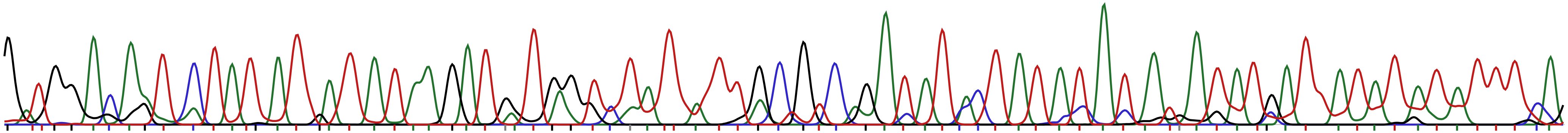

900 910 920 930 940 950 960 970

G T T G G A C A G T C T A T G A T G A T A T A A G A T - A T G G T C - A T T A T T G C G T C G A T A T C C T A T A T A T A T - A T A T G A T A T A T A T A T T T C A

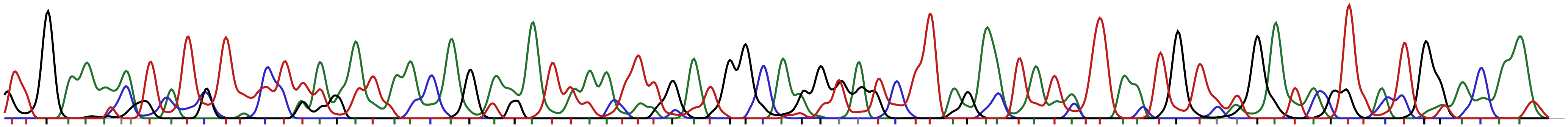

980 990 1000 1010 1020 1030 1040 1050

T T G A A A - - T A T C T T C T T A G A T A A C A G A G A T T A A T T T G A T G G C A G G - - T C T T A G A A T A T A T T A A T G T - - G A T A G T T C T G G A C A A
